# A new role for an old player: The rabies virus phosphoprotein as an RNA chaperone, helicase, and immune evasion hub

**DOI:** 10.1016/j.omtn.2026.103008

**Published:** 2026-08-07

**Authors:** Mohamed A. Hammad, Hossam M. Ashour

**Affiliations:** 1 Department of Medical Oncology, UH Seidman Cancer Center, University Hospitals and Case Western Reserve University, Cleveland, OH 44106, USA; 2 Department of Integrative Biology, College of Arts and Sciences, University of South Florida, St. Petersburg, FL, USA

## Main text

Rabies virus (RABV) remains one of the most lethal pathogens known, with a case-fatality rate approaching 100% once clinical symptoms appear. Despite effective post-exposure prophylaxis, thousands of deaths occur annually, and breakthrough infections remain a concern.^1^ The viral phosphoprotein (P) is a multifunctional hub essential for transcription, replication, and immune evasion, yet its full repertoire of activities has remained incompletely understood. The study by Mu and colleagues, published in the March issue of *Molecular Therapy Nucleic Acids*, uncovers a remarkable new facet of this protein: RABV-P possesses both NTP-dependent helicase-like and NTP-independent RNA chaperone activities, representing the first demonstration of such functions in the *Rhabdoviridae* family.^2^ This discovery reshapes our understanding of the viral life cycle and opens new therapeutic avenues.

The P protein of non-segmented negative-strand RNA viruses (NNSVs) has long been recognized as a multifunctional “scaffold” that coordinates the polymerase complex and chaperones the nucleoprotein.^3^ However, the absence of conserved helicase motifs in these proteins led to the assumption that they lacked intrinsic RNA remodeling capabilities. The exception was Ebola virus VP35, a functional analog of P proteins, which was shown to possess helicase-like activity.^4^ The structural and functional parallels between filoviral VP35 and rhabdoviral P proteins prompted Mu et al. to investigate whether RABV-P might similarly possess RNA-unwinding activity. Their findings confirm this hypothesis and extend it in unexpected ways, revealing that RABV-P is not merely a passive binding partner but an active participant in RNA remodeling.

The authors demonstrate that RABV-P exhibits robust NTPase activity with a preference for ATP and GTP and that this activity is divalent metal ion dependent. Importantly, the protein unwinds RNA duplexes in a bidirectional manner (both 5′→3′ and 3′→5′), a property that distinguishes it from EBOV VP35, which functions unidirectionally.^4^ This bidirectional activity, combined with the observation that RABV-P also possesses NTP-independent RNA unwinding and annealing activity, positions it as a bona fide RNA chaperone.^5^ The structural basis for these activities was mapped to the central oligomerization domain (COD) and the C-terminal domain (CTD), with the CTD being absolutely required for all unwinding functions and the COD being specifically critical for the NTP-dependent helicase-like activity.^6^ The authors further identified key residues R113 and K116 within the COD that are essential for ATPase activity, providing a molecular handle for understanding the enzymatic mechanism.

From a mechanistic perspective, these findings suggest a model in which RABV-P actively participates in resolving double-stranded RNA intermediates that arise during replication. The dsRNA replication intermediates must be unwound to liberate positive-sense RNA for translation or for use as a template for further replication and to allow negative-sense RNA to assemble into new ribonucleoprotein complexes. The rapid binding and dissociation kinetics observed in this study are consistent with the dynamic role of an RNA chaperone that cycles on and off RNA substrates to facilitate proper folding and template availability.^5^ Notably, the authors propose a model in which RABV-P uses its ATPase activity to energize dsRNA unwinding, while its NTP-independent activity may serve to resolve misfolded structures or facilitate strand annealing functions that are critical for the efficient flow of information during the viral life cycle.

**Figure 1:**
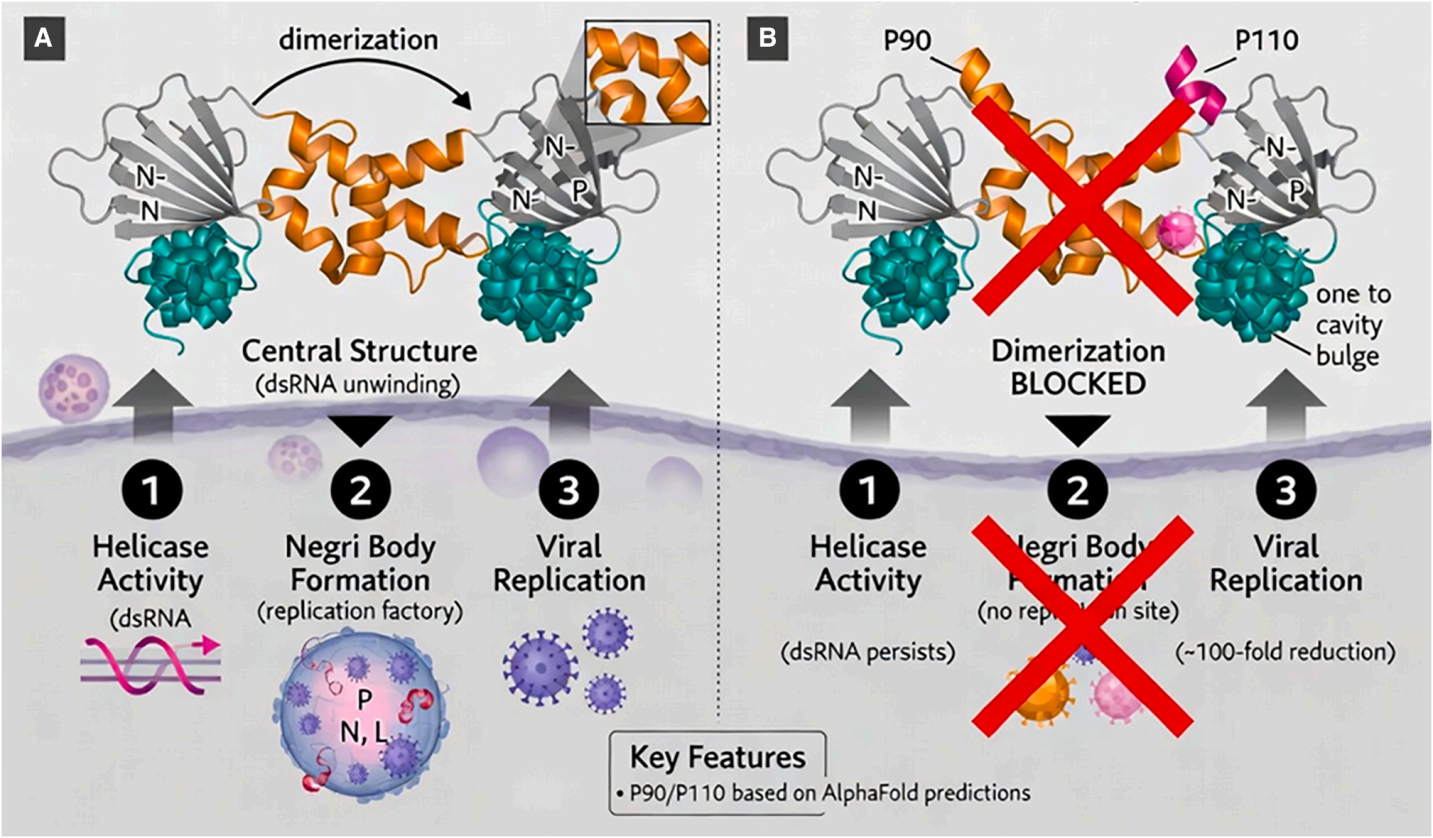
RABV-P dimerization is essential for helicase activity, Negri body formation, and viral replication, and is effectively blocked by designer peptides P90 and P110 (A) Normal pathway showing the three key steps enabled by P dimerization. (B) Peptide inhibition (P90 and P110) blocking dimerization and abolishing steps 1–3, resulting in ∼100-fold reduction in viral replication. EC_50_ ≈ 5.7 μM for both peptides; RABV specific.

The therapeutic implications of this work are particularly compelling. By designing peptides targeting the dimerization interface of the COD (P90 and P110), the authors demonstrate potent inhibition of RABV replication in cell culture, with a reduction in viral titer of up to 100-fold (Figure 1). These peptides appear to function by disrupting P dimerization, which in turn inhibits both its helicase activity and its ability to form Negri bodies (viral replication factories).^7^ This dual mechanism that targets both a structural function and an enzymatic activity makes P an attractive target for antiviral development. Moreover, the specificity of these peptides for RABV (with no activity against Zika virus or herpes simplex virus) suggests that they may act through a mechanism that is unique to the rhabdovirus P protein, potentially reducing off-target effects. The half maximal effective concentration (EC_50_) values of 5.7 μM for P90 are particularly encouraging and compare favorably with other antiviral candidates in development.

This discovery also raises important questions for future investigation. First, does the RNA-unwinding activity of RABV-P extend to other lyssaviruses or to P proteins of other NNSV families? Second, how does this activity integrate with the well-established roles of P in transcriptional regulation and immune evasion^8^? It is tempting to speculate that the ability to remodel RNA may facilitate the switch between transcription and replication or enable the virus to evade host RNA-sensing pathways. Third, can the peptide inhibitors be optimized for *in vivo* efficacy and developed into a viable therapeutic strategy for rabies, which currently lacks effective post-symptom treatments? The fact that P90 and P110 target COD, a region that may be structurally conserved across lyssaviruses, suggests potential for broad-spectrum activity within this genus. Fourth, what are the precise RNA substrates that RABV-P encounters during infection, and does the protein exhibit sequence or structural preferences that direct its activity to specific viral RNA elements? Understanding these preferences could guide the design of more selective inhibitors and reveal additional regulatory checkpoints in the viral life cycle. Fifth, does the NTP-independent unwinding activity play a distinct role *in vivo*, perhaps in resolving RNA structures that have become kinetically trapped during the crowded environment of the Negri body?

The study by Mu et al. represents a significant advance in our understanding of the RABV life cycle and opens new possibilities for antiviral development.^2^ The identification of RNA chaperone and helicase-like activities in a protein that was previously thought to function primarily as a structural scaffold illustrates the remarkable functional economy of viral proteins and highlights the need for continued exploration of their untapped enzymatic potential. As the field moves forward, structural determination of the P protein in complex with RNA substrates,^9^^,^^10^ as well as *in vivo* studies of peptide inhibitors, will be crucial for translating these findings into clinical applications. For a disease as deadly as rabies, every new therapeutic target is important, and the P protein now stands as one of the most promising candidates yet.^1^ The conceptual framework established here may extend beyond RABV, suggesting that RNA remodeling activities may be an underappreciated feature of P proteins across the NNSV order, potentially offering a unified therapeutic strategy for multiple deadly viruses. Given the recent development of peptidomimetics and stapled peptides that can overcome the bioavailability limitations of linear peptides, the path toward clinical translation of P-targeting inhibitors appears increasingly feasible.

Moreover, this work raises intriguing questions about the emergence of RNA remodeling activities in viruses that lack canonical helicase domains. The convergent development of such functions in phylogenetically distant viruses like filoviruses and rhabdoviruses suggests that RNA unwinding may be an essential but previously unrecognized requirement for NNSV replication (Table 1). This insight could reshape our understanding of the minimal viral replication machinery and may lead to the discovery of similar activities in other NNSV families, including paramyxoviruses and pneumoviruses, which cause significant human diseases such as measles and respiratory syncytial virus infection. The peptide-based approach described here provides a template for developing inhibitors against these related pathogens, expanding the therapeutic arsenal against deadly viral diseases.

**Table 1:** Comparative overview of RNA remodeling activities in NNSV proteins

Virus family	Virus	Protein	NTPase activity	Helicase-like activity	Directionality	NTP-independent activity
*Filoviridae*	Ebola virus	VP35	✓	✓	5′→3′ only	✗
*Rhabdoviridae*	rabies virus	P ^1^	✓	✓	bidirectional (5′→3′ & 3′→5′)	✓ (unwinding and annealing)
*Paramyxoviridae*	measles virus	P	unknown	unknown	unknown	unknown
*Paramyxoviridae*	Sendai virus	P	unknown	unknown	unknown	unknown
Pneumoviridae	RSV	P	unknown	unknown	unknown	unknown

## Author contributions

M.A.H. and H.M.A. contributed to the conceptualization, design, and visualization of this commentary. Both authors participated in writing the original draft, reviewing, and editing the manuscript, and H.M.A. provided project administration and supervision.

## Declaration of interests

The authors declare no competing interests.

## References

[1] Mu J., Wang C., Shu T., Xiong X., Ren Y., Gong R., Zhao L., Zhou X. (2026). Rabies lyssavirus phosphoprotein possesses RNA duplex-unwinding activities. Mol. Ther. Nucleic Acids.

[2] Shu T., Gan T., Bai P., Wang X., Qian Q., Zhou H., Cheng Q., Qiu Y., Yin L., Zhong J., Zhou X. (2019). Ebola virus VP35 has novel NTPase and helicase-like activities. Nucleic Acids Res..

[3] Ivanov I., Crépin T., Jamin M., Ruigrok R.W.H. (2010). Structure of the dimerization domain of the rabies virus phosphoprotein. J. Virol..

[4] Brzózka K., Finke S., Conzelmann K.K. (2005). Identification of the rabies virus alpha/beta interferon antagonist: Phosphoprotein P interferes with phosphorylation of interferon regulatory factor 3. J. Virol..

[5] Lahaye X., Vidy A., Pomier C., Obiang L., Harper F., Gaudin Y., Blondel D. (2009). Functional characterization of Negri bodies (NBs) in rabies virus-infected cells: Evidence that NBs are sites of viral transcription and replication. J. Virol..

[6] Jarmoskaite I., Russell R. (2014). RNA Helicase Proteins as Chaperones and Remodelers. Annu. Rev. Biochem..

[7] Fooks A.R., Cliquet F., Finke S., Freuling C., Hemachudha T., Mani R.S., Müller T., Nadin-Davis S., Picard-Meyer E., Wilde H., Banyard A.C. (2017). Rabies. Nat. Rev. Dis. Primers.

[8] Mavrakis M., Méhouas S., Réal E., Iseni F., Blondel D., Tordo N., Ruigrok R.W.H. (2006). Rabies virus chaperone: Identification of the phosphoprotein peptide that keeps nucleoprotein soluble and free from non-specific RNA. Virology.

[9] Albertini A.A.V., Wernimont A.K., Muziol T., Ravelli R.B.G., Clapier C.R., Schoehn G., Weissenhorn W., Ruigrok R.W.H. (2006). Crystal structure of the rabies virus nucleoprotein-RNA complex. Science.

[10] Gérard F.C.A., Bourhis J.M., Mas C., Branchard A., Vu D.D., Varhoshkova S., Leyrat C., Jamin M. (2022). Structure and Dynamics of the Unassembled Nucleoprotein of Rabies Virus in Complex with Its Phosphoprotein Chaperone Module. Viruses.

